# Acute Respiratory Failure of Unknown Etiology After Charcoal-Burning Suicide Attempt: Suspected Hypersensitivity Pneumonitis

**DOI:** 10.7759/cureus.40238

**Published:** 2023-06-10

**Authors:** Masaatsu Kuwahara, Hiroko Otagaki, Hideaki Imanaka

**Affiliations:** 1 Department of Emergency Medicine, Takarazuka City Hospital, Takarazuka, JPN

**Keywords:** respiratory failure, pneumonia, extrinsic allergic alveolitis, charcoal, attempted suicide

## Abstract

We report a case of a 25-year-old man who presented to the emergency department with respiratory distress after attempting suicide using burning charcoal briquettes. Charcoal briquette suicide is a method of suicide by carbon monoxide poisoning through inhalation of carbon monoxide produced when charcoal briquettes are burned.

The patient had a history of childhood asthma, but he was not on any scheduled treatment regimen. Upon admission, he had an elevated respiratory rate, hypoxic respiratory failure, and bilateral respiratory wheezing. Computed tomography showed significant mottled and infiltrated shadows in the upper lobes of both lungs, and hypersensitivity pneumonitis was suspected. Sputum culture, autoantibodies such as antinuclear antibodies, and other diagnostic tests ruled out other conditions. The patient was treated with antibacterial agents and steroids. Imaging tests showed improvement over time. He was discharged on the seventh day.

Charcoal briquette is a rare antigen that can potentially trigger hypersensitivity pneumonitis. Physicians should consider hypersensitivity pneumonitis as the differential diagnosis of respiratory failure after a charcoal-burning suicide attempt.

## Introduction

Charcoal briquette suicide is a common method of suicide in Asia, in which charcoal briquettes are burned in a confined space to produce carbon monoxide gas [1]. Burning charcoal briquettes has been associated with elevated levels of air pollutants such as PM2.5 and formaldehyde [2]. The major problem with burning charcoal briquettes is carbon monoxide poisoning. To date, there have been no reported cases of an association between charcoal briquettes and hypersensitivity pneumonitis (HP). HP is a complex interstitial lung disease caused by exposure to inhaled antigens from molds, birds, chemicals, or other sources [3]. We report a rare case of respiratory failure not caused by carbon monoxide poisoning after a charcoal briquette suicide attempt.

## Case presentation

A 25-year-old male patient presented to our hospital with a history of attempted suicide. He was a college student. It was the first time he tried to commit charcoal briquette suicide. He had been burning charcoal briquettes but was frightened and stopped halfway through. He had medium respiratory distress that night, for which he visited another emergency room but was sent home with only inhalation treatment (details unknown) for a suspected asthma attack. The patient had a history of childhood asthma for which he was not receiving any treatment. The patient was also a non-smoker.

The next morning (after about 10 hours), he continued experiencing breathing difficulties and visited our emergency department. His respiratory rate increased (32/min) and experienced worsening oxygenation (oxygen saturation of 90% on room air). He was conscious (Glasgow Coma Scale score: 4-5-6), his circulation was stable (blood pressure: 128/78; heart rate: 72 bpm), and his body temperature was elevated to 38.2°C. On chest auscultation, bilateral wheezing was present at the end of expiration.

Blood samples collected at admission showed elevated white blood cell counts and C-reactive protein levels (Table 1). Tests for severe acute respiratory syndrome coronavirus 2 (SARS-CoV-2) and influenza antigen were negative.

**Table 1:** Results of blood sampling at the time of admission TP: total protein; ALB: albumin; T-Bil: total bilirubin; AST: aspartate aminotransferase; ALT: alanine aminotransferase; LD: lactate dehydrogenase; UN: urea nitrogen; CRE: creatinine; Na: sodium; K: potassium; Cl: chloride; CRP: C-reactive protein; GLU: glucose; WBC: white blood cell count; Hb: hemoglobin; pCO2: partial pressure of carbon dioxide; pO2: partial pressure of oxygen; HCO3: bicarbonate; BE: base excess; Lac: lactate; KL-6: sialylated carbohydrate antigen KL-6.

Test	Result	Reference range
TP	7.1	6.6–8.1 g/dL
ALB	4.4	4.1–5.1 g/dL
T-Bil	3.5 H	0.4–1.5 mg/dL
AST	23	13–30 U/L
ALT	62 H	10–42 U/L
LD	169	124–222 U/L
UN	11.3	8–20 mg/dL
CRE	0.83	0.65–1.07 mg/dL
Na	142	138–145 mmol/L
K	4.1	3.6–4.8 mmol/L
Cl	104	101–108 mmol/L
CRP	3.22 H	<0.14 mg/dL
GLU	119 H	73–109 mg/dL
WBC	22.3 H	3.30–8.60 10^3/uL
Neutrophils	92.2	38–58%
Eosinophils	1.2	2–7%
Basophils	0.3	0–1%
Monocytes	3.2	3–8%
Lymphocytes	3	26–47%
Hb	16.5	13.7–16.8 g/dL
Platelet count	257	158–348 10^3/uL
KL-6	109	<500 U/ml
D-dimer	0.7	<1.0 μg/mL
pH	7.367	7.35–7.45
pCO2	44.6	35–45 mm Hg
pO2 (FiO_2_: 0.21)	41.7	80–100 mm Hg
HCO3	25	21–28 mmol/L
BE	-0.3	-2 to 3 mmol/L
Anion gap	12.5	10–20 mmol/L
Lac	33 H	4.5–14.4 mg/dL

However, the carboxyhemoglobin level was mildly elevated at 4.0% and the lactate level was as high as 33 mg/dl when the patient arrived at the hospital. Considering that the patient was a non-smoker, these results indicated that he exhibited symptoms consistent with carbon monoxide poisoning following the charcoal briquette suicide attempt the day before.

**Figure 1:**
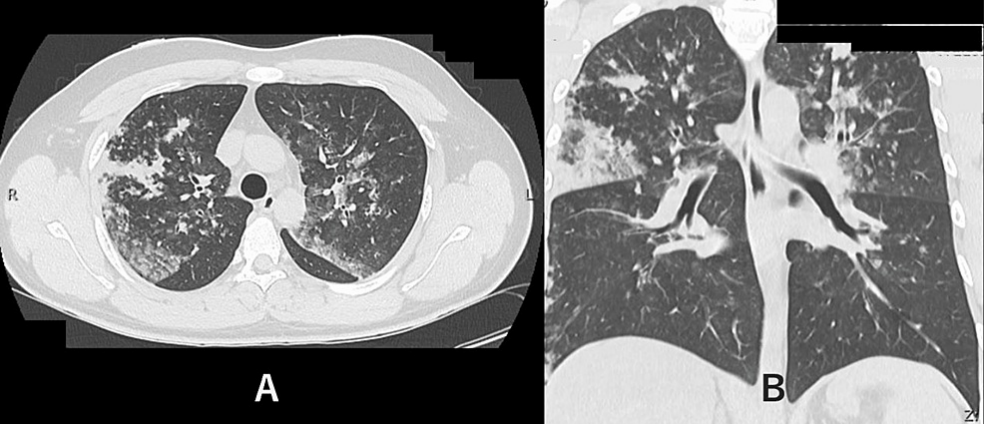
Chest computed tomography at the time of arrival A: axial view. B: coronal view.

A computed tomography (CT) scan of the chest showed significant mottled and infiltrated shadows, and mild bronchiectasis in the upper lobes of both lungs, and HP was suspected (Figure 1).

It was difficult to make a definitive diagnosis at this point because of many differentiating factors, such as infection, HP, and collagen lung disease. We initiated treatment with antibacterial agents (ceftriaxone, 2 g/day) and steroids (methylprednisolone, 125 mg twice a day) after performing sputum cultures and various autoantibody tests, including antinuclear antibodies and anti-neutrophil cytoplasmic antibodies. He was also started on oxygen at 3 l/min. Bronchoscopy was proposed, but the patient did not provide consent.

The sputum culture was negative for acid-fast bacilli and other bacteria. Moreover, the results of tests for collagen disease autoantibodies, influenza, and SARS-CoV-2 were negative. These results ruled out bacterial pneumonia, viral pneumonia, collagen lung disease, and tuberculosis. Considering the imaging diagnosis and clinical course of respiratory distress after the charcoal briquette suicide attempt, we have listed HP, acute lung injury, and acute respiratory distress syndrome as differentials.

**Figure 2:**
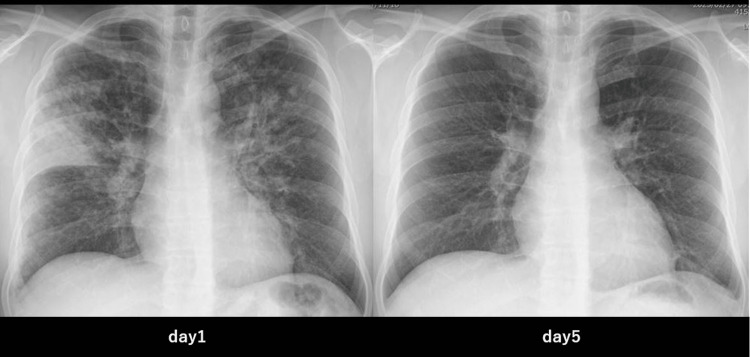
Chest X-ray images at the initial visit and after treatment

The patient responded to treatment, his respiratory distress improved, and oxygen administration was discontinued on the fifth day of hospitalization. Imaging tests also showed improvement over time, as shown in Figure 2.

Antibacterial drugs and steroids were administered for five days. The patient was discharged on the seventh day of hospitalization without any deterioration in his respiratory condition after completion of treatment. When he was discharged from the hospital, he did not require oxygenation. He was subsequently referred to a psychiatrist but refused inpatient treatment.

## Discussion

Herein, we report a case of respiratory distress and suspected HP following an attempted suicide by charcoal briquette consumption. Carbon monoxide poisoning is a well-known cause of respiratory failure due to charcoal briquette suicide, but other causes have not been widely reported.

The incidence of HP is reported to be approximately one in 100,000 people in the United Kingdom [4]. In the United States, an annual incidence rate of 1.7-2.7 per 100,000 people has recently been reported [5], and in Japan and France, the incidence rate is estimated at 0.3-0.9 per 100,000 people [6,7].

A list of antigens associated with HP and sources of exposure identified to date can be found in the American Thoracic Society, Japanese Respiratory Society, and Asociación Latinoamericana de Tórax guidelines [3]. The most frequent antigens are molds (most often caused by a mold called *Trichosporon*, among others). Other common antigens include a type of bacteria, proteins found in bird excrement, mushroom spores, and isocyanate, a raw material used to make polyurethane.

A differential diagnosis in the reported case could be pulmonary edema due to smoke inhalation. Smoke is a mixture comprising particulates, respiratory irritants, systemic toxins, and even heat, all of which contribute to a morbid insult. Moreover, exposure to wood smoke causes protein denaturation in the airway mucosa, induces inflammation, and damages the alveolar-capillary membrane and increases its permeability, thereby leading to pulmonary edema [8].

Burning biomass fuels, including charcoal briquettes, may increase lower respiratory tract infections in children and women [9,10].

In this case, the possibility that the patient had pulmonary edema cannot be ruled out. However, the fact that the patient was febrile when he came to our hospital could not be explained by pulmonary edema alone. The sputum culture was negative for acid-fast bacilli and other bacteria. Moreover, the results of tests for collagen disease autoantibodies, influenza, and SARS-CoV-2 were negative. These results ruled out bacterial pneumonia, viral pneumonia, collagen lung disease, and tuberculosis.

Bronchoscopy is useful in differentiating between HP and pulmonary edema. In this case, the patient presented with respiratory failure due to exposure to smoke from burning charcoal briquettes, and imaging findings were consistent with HP. In addition, the fact that the patient’s symptoms were relieved by steroid administration was also consistent with the possibility of HP. However, since bronchoscopy was not performed, the diagnosis of HP was considered to have moderate-level confidence [3].

A limitation of this case is that we could not obtain consent for bronchoscopy. We believe that bronchoscopy could have provided a more definitive diagnosis.

## Conclusions

Herein, we report a case of respiratory distress and suspected HP following an attempted suicide by charcoal briquette consumption. Emergency physicians should be aware that HP may be one of the differentials of respiratory distress after charcoal consumption suicide attempts.

## References

[1] Tsai CW, Gunnell D, Chou YH, Kuo CJ, Lee MB, Chen YY (2011). Why do people choose charcoal burning as a method of suicide? An interview based study of survivors in Taiwan. J Affect Disord.

[2] Huang HL, Lee WG, Wu FS (2016). Emissions of air pollutants from indoor charcoal barbecue. J Hazard Mater.

[3] Raghu G, Remy-Jardin M, Ryerson CJ (2020). Diagnosis of hypersensitivity pneumonitis in adults. An official ATS/JRS/ALAT clinical practice guideline. Am J Respir Crit Care Med.

[4] Solaymani-Dodaran M, West J, Smith C, Hubbard R (2007). Extrinsic allergic alveolitis: incidence and mortality in the general population. QJM.

[5] Fernández Pérez ER, Kong AM, Raimundo K, Koelsch TL, Kulkarni R, Cole AL (2018). Epidemiology of hypersensitivity pneumonitis among an insured population in the United States: a claims-based cohort analysis. Ann Am Thorac Soc.

[6] Yoshida K, Suga M, Nishiura Y, Arima K, Yoneda R, Tamura M, Ando M (1995). Occupational hypersensitivity pneumonitis in Japan: data on a nationwide epidemiological study. Occup Environ Med.

[7] Dalphin JC (1992). Extrinsic allergic alveolitis in agricultural environment. (Article in French). Rev Prat.

[8] Latenser BA, Iteld L (2001). Smoke inhalation injury. Semin Respir Crit Care Med.

[9] Mortimer K, Lesosky M, Semple S (2020). Pneumonia and exposure to household air pollution in children under the age of 5 years in rural Malawi: findings from the cooking and pneumonia study. Chest.

[10] Gordon SB, Bruce NG, Grigg J (2014). Respiratory risks from household air pollution in low and middle income countries. Lancet Respir Med.

